# Synthesis and Characterization of WO_3_/Graphene Nanocomposites for Enhanced Photocatalytic Activities by One-Step In-Situ Hydrothermal Reaction

**DOI:** 10.3390/ma11010147

**Published:** 2018-01-17

**Authors:** Xiaoxiao Hu, Peiquan Xu, Hongying Gong, Guotao Yin

**Affiliations:** College of Materials Engineering, Shanghai University of Engineering Science, Shanghai 201620, China; 18800233287@163.com (X.H.); ghyyw@163.com (H.G.); 18800236108@163.com (G.Y.)

**Keywords:** WO_3_/GR nanocomposites, hydrothermal method, photocatalysis, visible light

## Abstract

Tungsten trioxide (WO_3_) nanorods are synthesized on the surface of graphene (GR) sheets by using a one-step in-situ hydrothermal method employing sodium tungstate (Na_2_WO_4_·2H_2_O) and graphene oxide (GO) as precursors. The resulting WO_3_/GR nanocomposites are characterized by X-ray diffraction, Raman spectroscopy, transmission electron microscopy, scanning electron microscopy and X-ray photoelectron spectroscopy. The results confirm that the interface between WO_3_ nanorod and graphene contains chemical bonds. The enhanced optical absorption properties are measured by UV-vis diffuse reflectance spectra. The photocatalytic activity of the WO_3_/GR nanocomposites under visible light is evaluated by the photodegradation of methylene blue, where the degradation rate of WO_3_/GR nanocomposites is shown to be double that of pure WO_3_. This is attributed to the synergistic effect of graphene and the WO_3_ nanorod, which greatly enhances the photocatalytic performance of the prepared sample, reduces the recombination of the photogenerated electron-hole pairs and increases the visible light absorption efficiency. Finally, the photocatalytic mechanism of the WO_3_/GR nanocomposites is presented. The synthesis of the prepared sample is convenient, direct and environmentally friendly. The study reports a highly efficient composite photocatalyst for the degradation of contaminants that can be applied to cleaning up the environment.

## 1. Introduction

The use of semiconductor materials for the photocatalytic decomposition of organic pollutants in the gas or liquid states has aroused much interest from researchers [1,2,3]. Metal oxides, such as tungsten trioxide (WO_3_) and titanium dioxide (TiO_2_), and graphene are the most promising photocatalysts [4,5,6] owing to their high chemical stability, non-toxicity, and low cost. 

WO_3_, with a low band gap, is an important n-type semiconductor [7,8]. It has been widely used in gas sensing [9,10,11], lithium-ion batteries [12,13], smart windows [14] and photocatalysis [15,16]. WO_3_ exhibits both a high response and a high photocatalytic efficiency upon visible light irradiation [17]. However, pure WO_3_, as a photocatalyst, is not always beneficial because of the limitation of the electron–hole recombination rate and oxygen storage issues. Therefore, the following methods have previously been presented [18,19,20,21,22,23,24,25]: (1) changing the morphology of WO_3_ and the particle size; (2) loading a noble metal, doping metal or non-metal in WO_3_; and (3) combining with semiconductors. Liu et al. [26] prepared heterojunction of WO_3_/g-C_3_N_4_ with well-defined morphology by in-situ liquid phase process, and start materials metal-free g-C_3_N_4_ was synthesized by a thermal polycondensation method. The sample was obtained by heating in Ar atmosphere at 400 °C for 3 h. Dinari et al. [27] studied tungsten trioxide/multi-walled carbon nanotube system (MWCNT/WO_3_) as a novel photocatalyst by solvothermal process. The MWCNT/WO_3_ hybrid nanostructures exhibited higher photocatalytic activity than pure WO_3_ or MWCNTs due to their higher absorption enhancement in visible light region. The combination of graphene (GR) and semiconductor nanostructures has attracted great interest.

GR is a carbonaceous material with a two-dimensional honeycomb structure containing sp^2^ hybridized orbitals [1,28]. GR-based semiconductor photocatalysts for applications in the environment and energy have attracted considerable interest. For the first time, Williams et al. [29] obtained graphene oxide/TiO_2_ composites by ultrasonically dispersing TiO_2_ nanoparticles and graphene oxide in ethanol, and proposed the rationality about graphene as an electron transfer medium in grapheme/TiO_2_ composite photocatalyst. Fan et al. [30] prepared ZnO-graphene composite by hydrothermal reaction route at 200 °C for 12 h. The materials exhibited higher photocatalytic efficiency than P25 and ZnO, which was due to resilience to photocorrosion effects when ZnO hybridized with graphene. The bonding of WO_3_ and graphene has been reported [31,32,33,34,35]. WO_3_/GR nanocomposites can be prepared by electrostatic self-assembly [36,37], electrochemical [38], hydrothermal [39] and solvothermal [40] routes. Guo et al. [15] prepared WO_3_@Graphene composites by using a simple sonochemical method, in which the average particle size of the WO_3_ was controlled at about 12 nm on the graphene sheets. The materials with optimized structures was twice and 1.8 times as much as that for pure WO_3_ (ca. 186 µmol·L^−1^) and mixed-WO_3_/Graphene (ca. 214 µmol·L^−1^) when used as photocatalyst for water splitting. Wang et al. [41] synthesized the composites of graphene-WO_3_ by in situ hydrothermal method using (NH_4_)_5_H_5_·[H_2_(WO_4_)_6_] H_2_O and GO as precursors. Toward the photocatalytic reduction of CO_2_ into hydrocarbon fuels under visible-light irradiation, they demonstrated that the graphene can elevate the conduction band of WO_3_ by X-ray photoelectron spectroscopy (XPS) valence band spectra. Azimirad et al. [42] also prepared three-dimensional graphene foam-WO_3_ (3D GF-WO_3_) nanocomposite by chemical vapor deposition (CVD) on a nickel foam skeleton. The 3D GF-WO_3_ composite material was applied in photocatalytic degradation of Rhodamine B dye which showed an excellent photocatalytic performance than pure WO_3_ due to faster transferring the photoexcited electrons. Prabhu et al. [43] reported the synthesis of reduced graphene oxide-WO_3_ (rGO-WO_3_) nanostructured catalysts by wet chemistry followed by thermal decomposition method. The efficient photocatalytic degradation of MB by the prepared catalysts was achieved due to high adsorption capacity of the rGO-WO_3_ nanostructures.

Although the above results have meant that WO_3_/GR nanocomposites have been applied in many fields, it has been confirmed that the gap structure and interfacial charge transfer of semiconducting materials changes owing to the introduction of graphene. This knowledge provides a theoretical basis for the combination of GR and WO_3_ to obtain an ideal photocatalyst. It has not been specified whether the combination of graphene and tungsten trioxide in the WO_3_/GR nanocomposites prepared by these methods are chemically or physically bonded. Chemically bonded WO_3_/GR is of critical importance for determining the properties of the composite materials.

Our general purpose is to obtain WO_3_/GR nanocomposites by a one-step in-situ hydrothermal synthesis using sodium tungstate and oxidized graphene as precursors. The present study aims to determine the influence of graphene oxide on the microstructure, morphology and photocatalytic activity of the composites. HRTEM-EDS (High-resolution transmission electron microscopy–energy dispersive X-ray spectroscopy) is used to reveal the bonding mechanism of WO_3_ and graphene. It is confirmed that atomic bonding exists between WO_3_ and graphene in the WO_3_/GR nanocomposites. The WO_3_/GR nanocomposites exhibit an excellent photocatalytic performance.

## 2. Materials and Methods

### 2.1. Materials

Sodium tungsten dihydrate (Na_2_WO_4_·2H_2_O), oxalic acid (H_2_C_2_O_4_) and anhydrous sodium sulfate (Na_2_SO_4_) were purchased from Sinopharm (Shanghai, China). A graphene oxide (GO) aqueous dispersion (2 mg/mL) was purchased from Nanjing Nano Technology Co., Ltd. (Nanjing, China). The other solvents and the chemicals were of analytical grade and used as received without further purification. Deionized-distilled water (DDW) was prepared and used exclusively in this study.

### 2.2. Synthesis of WO_3_/GR Nanocomposites

**Scheme 1:**
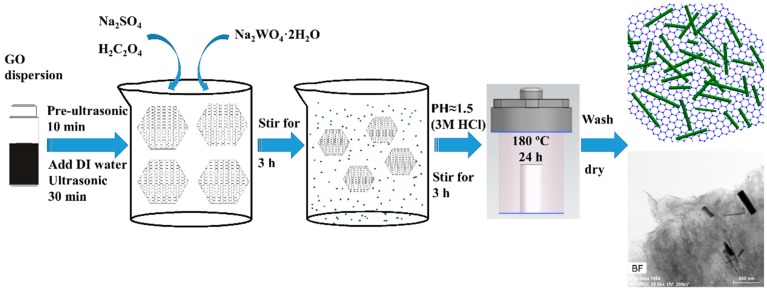
Flowchart for the preparation of WO_3_/GR (graphene) nanocomposites.

The WO_3_/GR nanocomposites were prepared by a one-step in-situ hydrothermal synthesis [44]. A flowchart for the WO_3_/GR nanocomposites preparation is shown in Scheme 1. Briefly, the stock GO dispersion (2 mg/mL) was pre-sonicated for 10 min and then the relevant GO dispersion (2 mL, 10 mL, and 20 mL) was dispersed in a certain amount of the deionized water, a total solution volume was 40 mL, in which the concentration of GO solution were 0.1 mg/mL, 0.5 mg/mL, and 1 mg/mL, respectively, then sonicated for 0.5 h at room temperature. Na_2_WO_4_·2H_2_O (0.5 g), H_2_C_2_O_4_ (1 g) and Na_2_SO_4_ (4 g) were added into the suspension and the resulting solution was stirred for 3 h at room temperature. With vigorous stirring, the pH of the solution was adjusted to 1.5 by adding 3M HCl, and then stirring was continued for 3 h. The solution was transferred to a Teflon-lined stainless-steel autoclave and kept at 180 °C for 24 h. The final mixture was filtered and washed (three times) with deionized water and absolute ethanol by cross-centrifugation. The residual liquid was placed in a dry oven at 60 °C for 5 h. A series of WO_3_/GR nanocomposites were prepared by varying the GO content. The samples were numbered as WO_3_/GR-*x* (*x* = 0.1, 0.5, and 1), where *x* represents the concentration of the aqueous solution of GO. Under the same experimental conditions, pure WO_3_ nanorods were prepared without GO for comparison with the obtained WO_3_/GR nanocomposites.

### 2.3. Characterizations of Materials

X-ray diffraction (XRD) was performed using an X’Pert PRO X-ray diffractometer (PANalytical B.V., Eindhoven, ZuidHolland, The Netherlands) with Cu K_α_ radiation (λ = 1.541 Å) in the 2θ range from 10° to 80° at a scan rate of 2°/min. Transmission electron microscopy (TEM) images of the WO_3_/GR nanocomposites were recorded using a FEI Titan Themis 200 TEM instrument (FEI, Hillsboro, OR, USA) operated at 200 kV equipped with an energy dispersive X-ray spectroscopy (EDS) detector (X-Max^N^ TSR, Oxford Instrument, Oxford, UK). Scanning electron microscopy (SEM) was conducted on a JSM-7500F (JEOL, Tokyo, Japan) microscope to examine the WO_3_/GR nanocomposites.

The chemical interactions and purity of the samples were characterized by Raman spectroscopy on a Raman spectrometer (Horiba Evolution, Paris, France), which was carried out at room temperature with a laser excitation source of 633 nm and a light spot size of 0.5 mm. X-ray photoelectron spectroscopy (XPS) was conducted on AXIS UltraDLD (Kratos, Kyoto, Japan) with an Mg K_α_ X-ray as the excitation source to determine the chemical information from the surface and interface of the sample. The optical absorption properties of the solid samples were characterized by UV-vis diffuse reflectance spectroscopy (DRS) on a UV-vis spectrophotometer (UV-3600, Shimadzu, Kyoto, Japan) with a sphere attachment and BaSO_4_ was used as the internal reflectance sample. 

### 2.4. Evaluation of Photocatalytic Activity

The photocatalytic activity experiments on the prepared samples were studied by examining the photodegradation reaction of methylene blue (MB) as an organic pollutant. In a typical reaction, the photocatalyst (20 mg) was added to an MB solution (50 mL, 10 mg·L^−1^). The solution was magnetically stirred in the dark for 1 h to achieve adsorption equilibrium. The resulting solution was then irradiated directly with a 300 W PLS-SXE 300 xenon lamp (Beijing Perfect Light Technology Co., Ltd., Beijing, China) equipped with a λ < 400 nm cut-off filter. The purpose of the cut-off filter was to remove wavelengths below 400 nm to ensure that only visible light (420–700 nm) was irradiating the sample. The distance between the light source and the sample level is 20 cm, the irradiated surface is 3 cm × 3 cm. A sample (5 mL) of the solution was extracted from the mixture every 15 min and centrifuged to remove solids. The centrifuged liquid was poured into a cuvette and its absorbance was measured by an ultraviolet spectrophotometer (UV-1900, Haida Scientific Co., Ltd., Shanghai, China). The maximum absorbance of MB at the wavelength of 664 nm was selected to analyze the change in concentration as the MB solution was degraded. The photocatalytic activity of the samples under visible light irradiation was determined.

## 3. Results and Discussion

**Figure 1:**
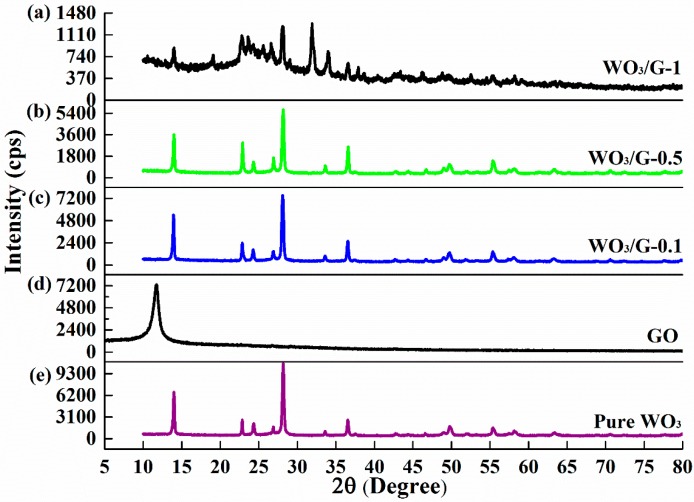
XRD patterns of pure WO_3_ and WO_3_/GR nanocomposites with different contents of GO. (**a**) WO_3_/G-1; (**b**) WO_3_/G-0.5; (**c**) WO_3_/G-0.1; (**d**) GO; (**e**) Pure WO_3_.

Figure 1 shows the XRD trace of WO_3_ and the WO_3_/GR nanocomposites doped with different amounts of GO. The XRD pattern of WO_3_ was composed of three strong lines and broad half peaks at the diffraction angles of 13.9°, 28.2° and 37.1°, which corresponded to the (100), (200) and (201) planes, respectively. This was consistent with the standard XRD pattern of hexagonal WO_3_. Hence, the sample contained hexagonal phase WO_3_. The sharpness of the main peaks indicated that the synthesized WO_3_ had a good degree of crystallinity.

The XRD patterns of the WO_3_/GR composites did not exhibit any special features compared with the XRD patterns of WO_3_. According to the literature [45,46,47], the characteristic peaks of graphene at 26° and 42° overlap with the WO_3_ peaks for the (101) and (300) planes. Typically, the characteristic peak of GO is at 11°. The absence of this peak confirmed that the GO had been reduced to graphene. As was mentioned above, it was again confirmed that hexagonal WO_3_ was successfully grown on graphene according to the XRD patterns. The presence of graphene could be further demonstrated by Raman measurements.

**Figure 2:**
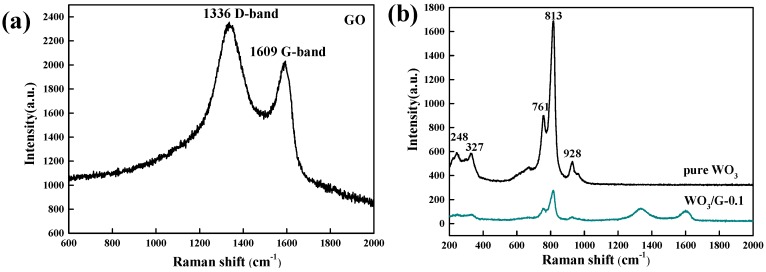
Raman spectra of: (**a**) GO; and (**b**) pure WO_3_ and WO_3_/GR-0.1 nanocomposite.

A selected region of the Raman spectra of GO is shown in Figure 2a. GO showed two peaks at approximately 1336 cm^−1^ and 1609 cm^−1^, the D-band and G-band, respectively, which was consistent with the results in the literature [48,49,50]. The G-band peak at around 1609 cm^−1^ is characteristic of a graphitic sheet corresponding to a well-defined sp^2^ carbon-type structure, which can be attributed to the doubly degenerate zone center E_2g_ mode [51], whereas the D-band at approximately 1336 cm^−1^ can be attributed to the presence of defects or edges within the hexagonal graphite structure.

Raman spectroscopy further characterized the molecular structure of the WO_3_/GR nanocomposites, as shown in Figure 2b. Peaks at 248 cm^−1^, 327 cm^−1^, 761 cm^−1^, 813 cm^−1^ and 928 cm^−1^ were observed in the Raman spectra of pure WO_3_ and the WO_3_/GR-0.1 nanocomposite. Among them, the peaks centered at 248 cm^−1^ and 327 cm^−1^ were attributed to the O–W–O bending mode (δ (O–W–O)), while those at 668 cm^−1^, 761 cm^−1^ and 813 cm^−1^ were attributed to the stretching vibration of tungsten atoms and adjacent oxygen atoms (ν (O–W–O)). The peak at 928 cm^−1^ arose from the symmetric stretching mode of the terminal W=O bond [υs (W=O)], which was consistent with the literature [15,31,32,33,34]. Compared with pure WO_3_, the peak intensity of the WO_3_ in the WO_3_/GR-0.1 nanocomposite was significantly weakened and broadened owing to the strong bonds between WO_3_ and graphene rather than the physical bonding (which weakens the internal W–O bond) as discussed by O. Akhavan et al. [52]. Two characteristic peaks at 1331 cm^−1^ and 1592 cm^−1^ were observed in the Raman spectrum of the WO_3_/GR-0.1 nanocomposite. The existence of graphene in the composites was confirmed. The characteristic peak of graphene tended to migrate slightly to a lower frequency. The intensity ratio I_D_/I_G_ between the band-D and band-G can be used to determine the extent of graphene material defects [15]. This value was calculated to be 1.27 in GO and 1.05 in the WO_3_/GR nanocomposites. The results indicated the absence of defects in the composites and many oxygen-containing functional groups were reduced during the hydrothermal process.

**Figure 3:**
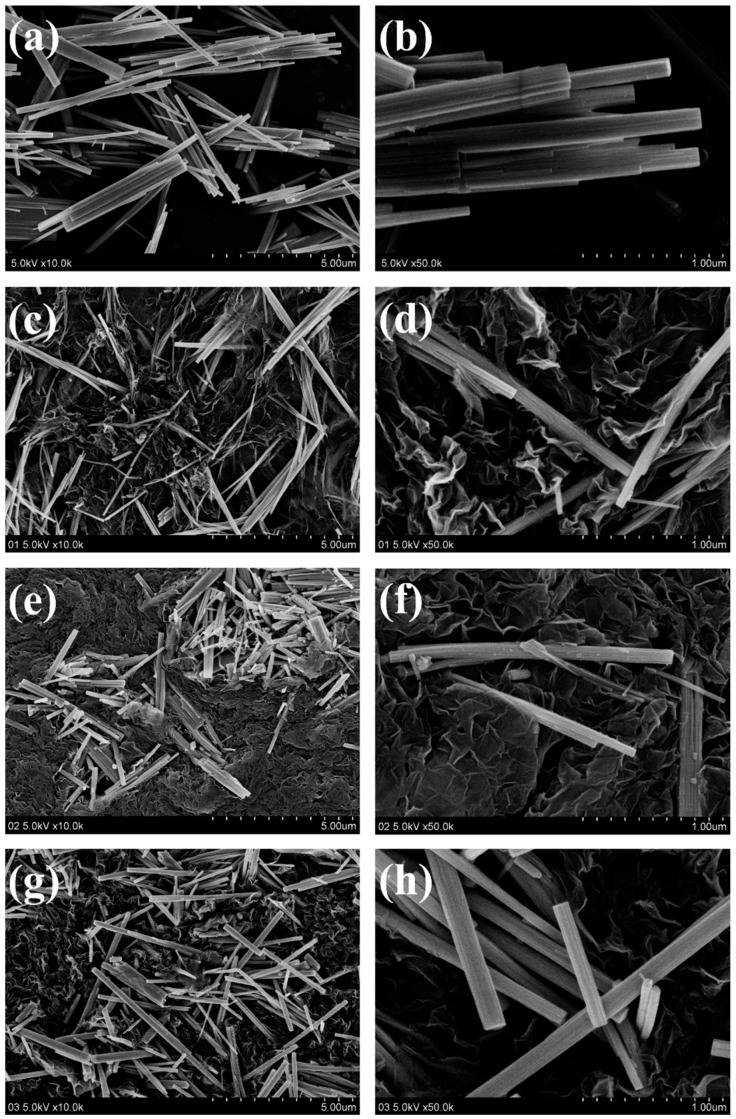
SEM images of: (**a**,**b**) WO_3_; (**c**,**d**) WO_3_/GR-0.1; (**e**,**f**) WO_3_/GR-0.5; and (**g**,**h**) WO_3_/GR-1.

**Figure 4:**
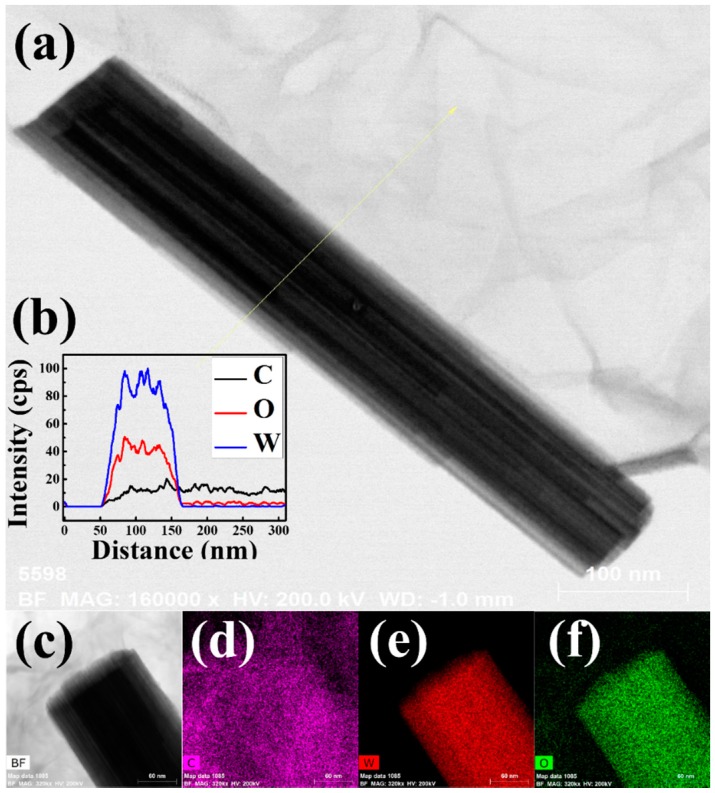
(**a**) Bright-field TEM images of WO_3_/GR-0.1 (the yellow area indicates the line-diffusion analysis position); and (**b**) EDS curves showing C, O and W elements in the direction of the arrow indicated in (**a**); (**c**) Bright-field TEM image of a select area; and (**d**–**f**) EDS element mapping images of C, O and W.

Figure 3a,b illustrates the microstructure of the WO_3_ nanorod. In the microstructures of the WO_3_/GR nanocomposites, WO_3_ nanorods were shown to be distributed on the surface of graphene at low magnification (Figure 3c,e,g) and high magnification (Figure 3d,f,h). A reunion phenomenon was observed for the WO_3_/GR-0.1 nanocomposite. At a high magnification, the graphene nanosheets were shown to have rough surfaces and curled edges. Similarly, agglomeration was still observed in the WO_3_/GR-0.5 nanocomposite. However, the amount of WO_3_ agglomeration significantly decreased with discoid WO_3_ at a higher magnification. The agglomeration of the WO_3_/GR-0.5 nanocomposite was not observed in the WO_3_/GR-1 nanocomposite. In the SEM images in Figure 3, the graphene was composed of a multi-GR sheets folded structure. It was concluded that the agglomeration of WO_3_ nanorods decreased with the increase of GO content. Therefore, the introduction of the appropriate amount of graphene can easily disperse the graphene sheets and inhibit the agglomeration of the WO_3_ nanorods, which increased the specific surface area and the active sites of the photocatalytic process and further improved the photocatalytic activity. An amount of WO_3_ nanorods was found to grow on the surface of graphene. This was further proven by the bright-field TEM images (Figure 4a) of the WO_3_/GR-0.1 nanocomposite, EDS curve (Figure 4b) through the WO_3_ nanorods and the EDS element mapping images of C, O and W (Figure 4d–f) in the selected area. As shown in the typical bright-field TEM image of the WO_3_/GR-0.1 nanocomposite (Figure 4c), the WO_3_ nanorods were fixed to the flocculated graphene. It was found that chemical bonds were formed between WO_3_ and the graphene sheets.

**Figure 5:**
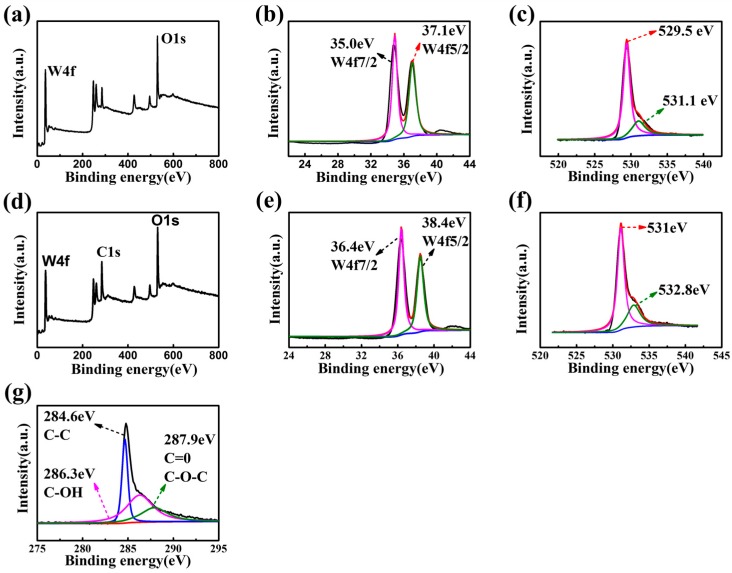
(**a**) The XPS full spectrum; and X-ray photoelectron spectra of: (**b**) W4f; and (**c**) O1s for pure WO_3_; (**d**) The XPS full spectrum; and X-ray photoelectron spectra of: (**e**) W4f; (**f**) O1s; and (**g**) C1s for the WO_3_/GR-0.1 nanocomposite.

As a powerful surface analysis technique, XPS is often used to determine the surface elemental composition, valence states and molecular structure of as-prepared samples. Figure 5 shows the XPS results of pure WO_3_ and the WO_3_/GR-0.1 nanocomposite. The elements of C, O and W could be easily identified through the characteristic peaks of C1s, O1s and W4f. The survey spectra of pure WO_3_ (Figure 5a) and the WO_3_/GR-0.1 nanocomposite (Figure 5d) exhibited similar peak shapes; therefore, the sample needed narrower scanning. The high-resolution W4f spectrum for pure WO_3_ could be deconvoluted into two peaks at 35.0 eV and 37.1 eV, which were assigned to the W4f7/2 and W4f5/2 spin orbital splitting photoelectrons of the W^6+^ chemical state. O1s can be decomposed into two separate binding energies at 529.5 eV and 531.1 eV, corresponding to the surface lattice oxygen (O_latt_) and surface adsorbed oxygen (O_ads_) [53], respectively (Figure 5b,c). In the WO_3_/GR-0.1 nanocomposites, the high-resolution spectrum of W4f are located at the binding energies of 36.4 eV and 38.4 eV, corresponding to the W4f7/2 and W4f5/2 peaks of the WO_3_ phase in the composite, respectively. Peak area ratio of these two peaks is obtained 0.75, which is in line with the 4f level theory of spin orbit splitting; its O1s XPS spectra can be deconvoluted into binding energy at 531 eV and 532.8 eV peaks, corresponding to the W–O bond and C–OH bond. In conclusion, the deconvolution signal of the binding energies of O1s and W4f in WO_3_/GR-0.1 composites is more intense and migrates to higher binding energy than pure WO_3_, it is probably due to the internal C, O and W elements of the interaction between the graphene and WO_3_, which is consistent with the results of Raman analysis. Figure 4 and Figure 5g is a high-resolution C1s spectrum of WO_3_/GR-0.1 nanocomposite; the three peaks are located at 284.6 eV, 286.3 eV and 287.9 eV binding energies, attributed to C–C, C–OH, C=O and C–O–C bonds, respectively.

**Figure 6:**
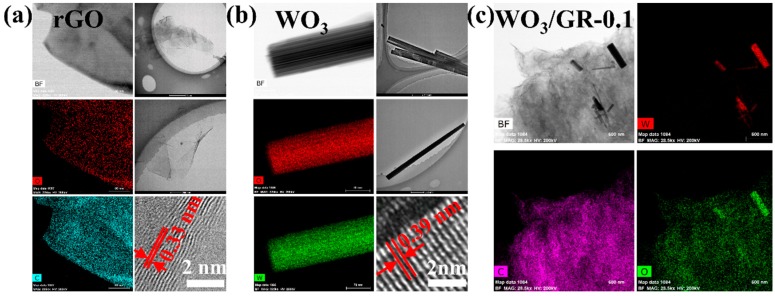
Bright-field TEM-EDS showing the microstructure and composition of: (**a**) rGO; (**b**) pure WO_3_; and (**c**) the WO_3_/GR-0.1 nanocomposite.

The representative TEM-EDS of rGO, WO_3_ and the WO_3_/GR-0.1 nanocomposite, and the corresponding HRTEM (High-resolution transmission electron microscopy) images are shown in Figure 6. As shown in Figure 6a, rGO is a two-dimensional nanosheet with several crinkles. The d-spacing of the lattice plane (002)_GR_ was approximately 0.33 nm. The typical bright-field TEM images (Figure 6b) demonstrated that WO_3_ presented as irregular nanorods. The irregular WO_3_ nanorods were assembled by radial multi-rods, among which the interstitial space could be observed if the content of WO_3_ was sufficiently large. The d-spacing of the lattice plane (001)_WO3_ was approximately 0.39 nm, which was in accordance with the (001)-spacing of h-WO_3_ crystals. The TEM image of WO_3_/GR nanocomposites is shown in Figure 6c, from which a hybrid nanoarchitecture with WO_3_ nanorods on the surface of graphene could be observed. The nanocomposites exhibited a clear interface between the WO_3_ nanorods and graphene nanosheets.

**Figure 7:**
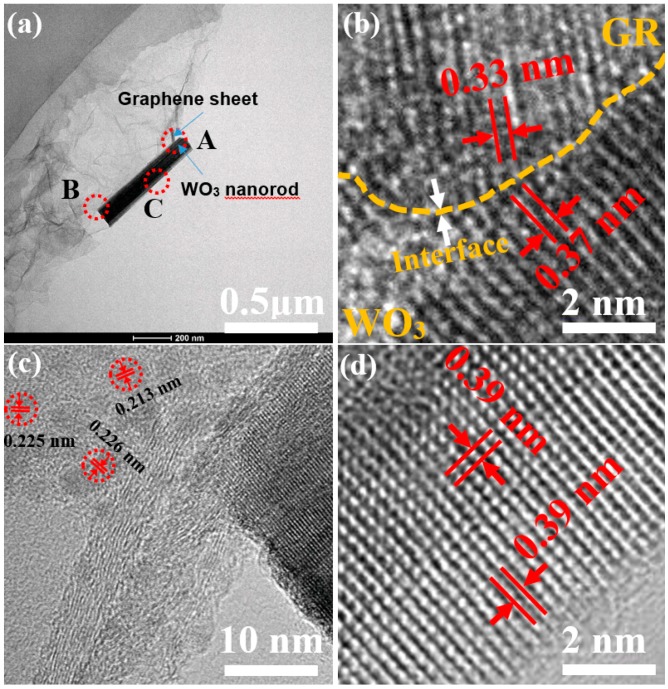
(**a**) Bright-field TEM images showing the WO_3_ nanorod on the graphene sheet in the WO_3_/GR-0.1 nanocomposite; HRTEM images of regions: (**b**) A; and (**c**) B showing the GR/WO_3_ interface; and (**d**) HRTEM image inside WO_3_ (region C in (**a**)).

Figure 7 reveals the morphology of the WO_3_ nanorod and the WO_3_/GR nanocomposites. As shown in Figure 7a, it was clear that the characteristic WO_3_ nanorods were successfully synthesized through a one-step in-situ hydrothermal reaction. Taking the WO_3_/GR-0.1 nanocomposite as an example, a one-dimensional WO_3_ nanorod was spread on the surface of the two-dimensional graphene nanosheet. The HRTEM images of regions B and C (Figure 7b,c) show the interfaces of the WO_3_ nanorod and graphene nanosheet. The results indicated that an intimate interfacial contact between them was readily synthesized by such a simple hydrothermal reaction approach. The d-spacing of the lattice plane (001) near the interface in Figure 7b became smaller as the interface was approached. Some stacking faults and dislocations were also observed at the interface. The HRTEM image of region C (Figure 7c) shows the different lattice structures around the interface between WO_3_ and graphene. The d-spacing of these lattice planes was in the range of 2.13 Å to 2.26 Å. Figure 7d shows the standard lattice plane (001). The result means that the lattice structure inside WO_3_ was not affected by the graphene or the interfacial energy.

**Figure 8:**
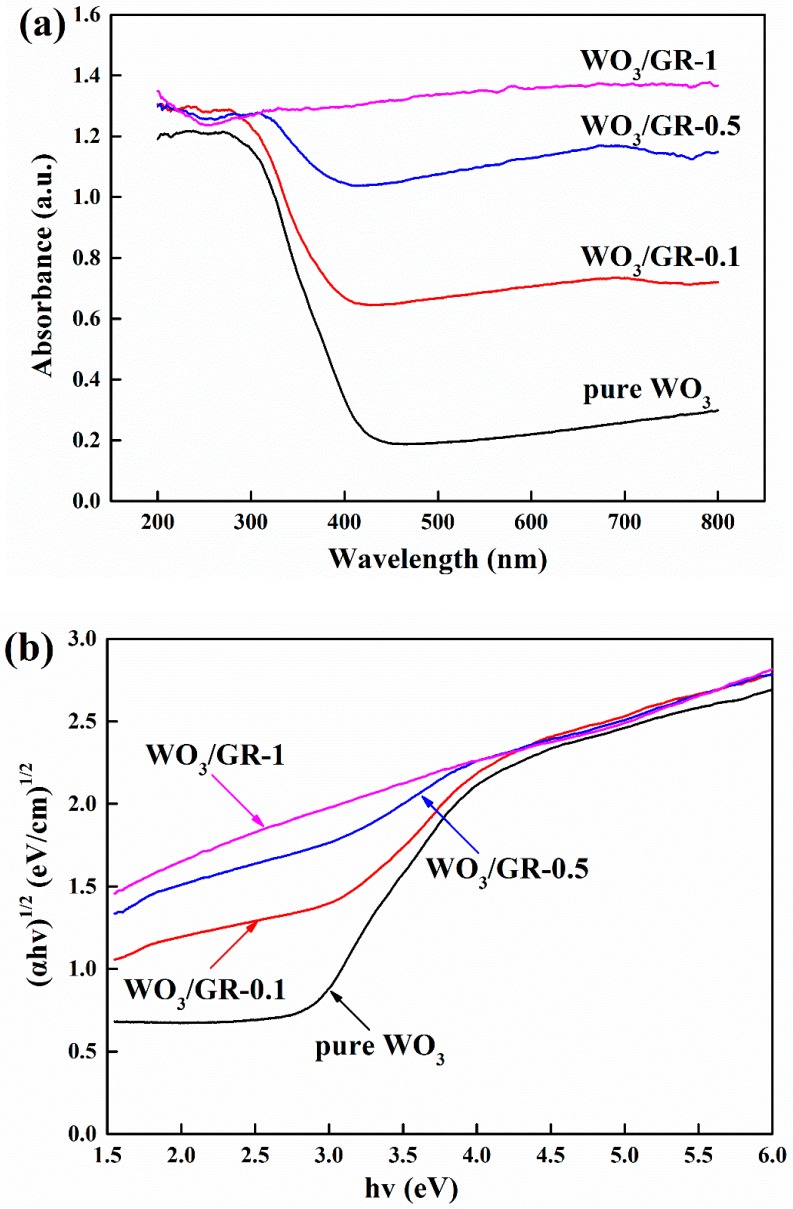
(**a**) UV–vis diffuse reflectance electronic spectra of pure WO_3_ and the WO_3_/GR nanocomposites; and (**b**) the relation curves of h*v* and corresponding (αh*v*)^1/2^ of the as-prepared samples.

Figure 8 shows the UV-vis diffuse reflectance electronic spectra of pure WO_3_, the WO_3_/GR nanocomposites with different contents of GO and the effect of h*v* on the corresponding (αh*v*)^1/2^ for the as-prepared samples. Compared with the pure WO_3_ nanorods, the WO_3_/GR nanocomposites showed a stronger photoabsorbance in the visible region from λ = 400 to 800 nm. This arose from the introduction of graphene. The solar light harvesting could be ascribed to the formation of W–O–C bonds between the WO_3_ and graphene [54]. In addition, the absorption edges were at 440 nm, 420 nm, 400 nm, and 255 nm for the pure WO_3_ nanorods, and the WO_3_/GR-0.1, WO_3_/GR-0.5, and WO_3_/GR-1 nanocomposites, respectively. It was clear that the absorption edge shifted to shorter wavelength for the WO_3_/GR nanocomposites with the increase of GO content. According to the following formula for the semiconductor band gap, the band gap of the sample can be calculated:

(1) $${\left(\frac{\alpha hv}{K}\right)}^{1/2}=\;hv-\mathrm{Eg}$$

where *K* = b × c, b is the thickness of the cuvette or film sample, and c is the concentration. *K* has no effect on Eg, α is the absorbance, h*v* is the energy of the exciton, and Eg is the band gap energy. Therefore, the band gap energies were 2.60, 2.25, 1.65 and 1.17 for pure WO_3_, and the WO_3_/GR-0.1, WO_3_/GR-0.5, and WO_3_/GR-1 nanocomposites, respectively.

**Figure 9:**
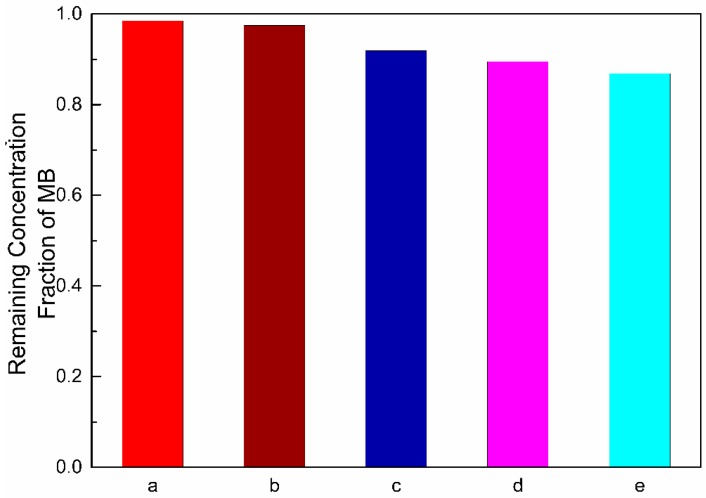
The remaining methylene blue (MB) in solution equilibrated with: (**a**) no catalyst; (**b**) pure WO_3_; (**c**) WO_3_/GR-0.1; (**d**) WO_3_/GR-0.5; and (**e**) WO_3_/GR-1 after stirring in the dark for 60 min.

As shown in Figure 9, the initial concentration of MB exhibited almost no changes in the catalyst-free and pure WO_3_ solutions, whereas a decrease in the solution containing WO_3_/GR nanocomposites arose from the introduction of graphene. Part of the dye molecule adsorbed onto the surface of the WO_3_/GR nanocomposites, which resulted in a decrease of the MB concentration after the adsorption equilibrium. This was also beneficial for enhancing the photocatalytic performance of the WO_3_/GR nanocomposites. 

**Figure 10:**
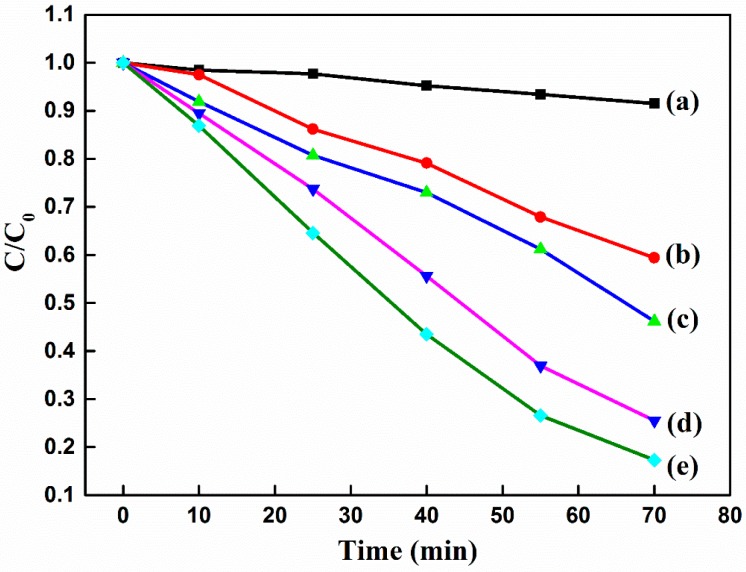
Photodegradation of MB under visible light: (**a**) no catalyst; (**b**) pure WO_3_; (**c**) WO_3_/GR-0.1; (**d**) WO_3_/GR-0.5; and (**e**) WO_3_/GR-1.

Figure 10 displays the curve of the normalized concentration (C/C_0_) of MB versus time under visible light, where C_0_ is the initial concentration of MB in solution, and C is the concentration of MB remaining in the solution at each illumination interval. After 70 min of illumination, the normalized concentrations of MB in solution were 92%, 59%, 46%, 26% and 17%, respectively, for the catalyst-free, pure WO_3_, and the WO_3_/G-0.1, WO_3_/G-0.5 and WO_3_/G-1 nanocomposites. Therefore, the WO_3_/GR composites exhibited a higher photocatalytic efficiency than pure WO_3_. With the increase of graphene oxide content, the photocatalytic efficiency of WO_3_/GR was also increased, which arose from the introduction of graphene. The introduction of graphene increased the optical absorption efficiency of the composite materials, which was consistent with the DRS results, and inhibited the recombination rate of photogenerated electron–hole pairs, which thus improved the photocatalytic efficiency. The maximum degradation rate was achieved by WO_3_/G-1 (83%), which was double that of pure WO_3_ (41%).

As mentioned above, some WO_3_-based and graphene-based composites had been prepared for the degradation of organic pollutants [26,27,42,43,55]. As shown in Table 1, their photocatalytic efficiency is usually 65–95% within 56–150 min. Compared with the previously reported composites used in the field of photocatalytic degradation of pollutants, WO_3_/GR prepared in this work shows a high photocatalytic efficiency within short time, which can be easily found in Table 1. In addition, the method of the composite is also simpler and more feasible.

**Figure 11:**
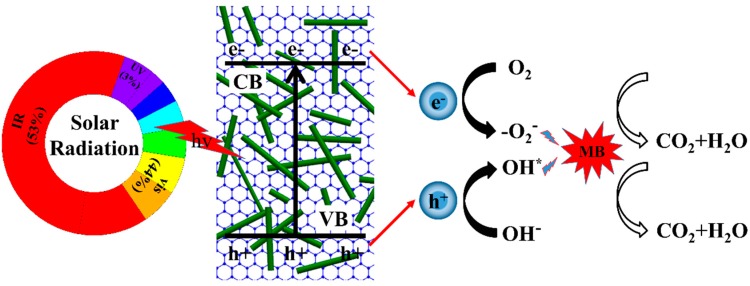
Schematic representation of photocatalytic degradation of methylene blue by WO_3_/GR nanocomposites under visible light illumination.

Figure 11 illustrates the mechanism of the photocatalytic degradation of MB by the WO_3_/GR nanocomposites under visible light irradiation. In the solar spectrum, the energy distribution in the ultraviolet spectrum almost disappears (UV light below 0.29 m is almost all absorbed), and only approximately 3% remains. The infrared spectrum occupies 53%, while the visible spectrum accounts for 44%, which is nearly half of the solar energy. Under visible light irradiation, h*v* (vis) ≥ Eg. The electron in WO_3_ acquires enough energy to generate electron–hole pairs; consequently, the WO_3_ electron in the valence band is transferred into the conduction band of graphene. These electron–hole pairs undergo reduction-oxidation reactions, where the valence band hole is a good oxidant and the electron in the conduction band is a good reductant. Owing to the high carrier mobility of graphene (20,000 cm^2^ V^−1^ s^−1^) and its 2D conjugated structure, the introduction of graphene behaves as an electron acceptor. Therefore, the efficiency of the electronic separation is enhanced. The rate of recombination of the photogenerated electron–hole pairs is inhibited. This results in an improvement of the photocatalytic efficiency for the WO_3_/GR nanocomposites. The process can be written as:

(2) $${\mathrm{WO}}_{3}/\mathrm{graphene}+\;hv\left(\mathrm{Vis}-\mathrm{irradiated}\right)\to e^{-}+{\mathrm{WO}}_{3}\left(h^{+}\right)$$

(3) $$O_{2}\left(\mathrm{absorbate}\right)+e^{-}\to O_{2}^{-}$$

(4) $$O_{2}\left(\mathrm{absorbate}\right)+2H_{2}O+e^{-}\to H_{2}O_{2}+2{\mathrm{OH}}^{-}$$

(5) $$H_{2}O_{2}+e^{-}\to {\mathrm{OH}}^{-}+{\mathrm{OH}}^{*}$$

(6) $${\mathrm{WO}}_{3}\left(h^{+}\right)+{\mathrm{OH}}^{-}\to {\mathrm{WO}}_{3}+{\mathrm{OH}}^{*}$$

(7) $${\mathrm{WO}}_{3}\left(h^{+}\right)+H_{2}O\to {\mathrm{WO}}_{3}+H^{+}+{\mathrm{OH}}^{*}$$

(8) $${\mathrm{OH}}^{*}+\mathrm{MB}\to {\mathrm{CO}}_{2}+H_{2}O$$

(9) $$O_{2}^{-}+\mathrm{MB}\to {\mathrm{CO}}_{2}+H_{2}O$$

## 4. Conclusions

WO_3_/GR nanocomposites, which are visible light photocatalysts, were successfully synthesized by a one-step in-situ hydrothermal method. This method could directly reduce GO to graphene without any reductant, and this was accompanied by the attachment to WO_3_. The formation of WO_3_/GR composites was confirmed by SEM, TEM, EDS, Raman and XPS. The presence of graphene in the composites promoted the electron transfer and optical absorption properties. The results showed that the WO_3_/GR composites exhibited an enhanced photocatalysis efficiency in visible light, which was double that of pure WO_3_. In addition, the synthesis method of the graphene-based composite material can be extended to other functional material synthesis fields to realize promising applications in photocatalysis.

## Figures and Tables

**Table 1:** Comparison of photocatalytic performance between this work and reported references.

Photocatalyst	Methods of Synthesis	Experimental Conditions	Photodecomposition	Photocatalytic Efficiency	References
WO_3_/GR	one-step in-situ hydrothermal reaction	Precursor: Na_2_WO_4_·2H_2_O and GO the structure-directing agent: H_2_C_2_O_4_ and Na_2_SO_4_ Time: 24 h Temp:180 °C	Degradation of methylene blue	Degradation percentage of 83% within 70 min	This work
3D graphene foam-WO_3_	chemical vapor deposition(CVD) (two steps)	Precursor: the nickel foam, CH_4_, tungsten wire Time: >54 h Temp: -	degradation of Rhodamine B dye	Degradation percentage of 85% within 140 min	[42]
rGO-WO_3_	wet chemistry and thermal decomposition method	Precursor: GO and H_2_WO_4_	Degradation of methylene blue	Degradation percentage of 65% within 60 min	[43]
WO_3_/g-C_3_N_4_	in-situ liquid phase process and heat treatment	Precursor: ammonium metatungstate hydrate and g-C_3_N_4_ the structure-directing agent: oxalic acid-ethanol solution	Degradation of methylene blue	Degradation percentage of 95% within 90 min	[26]
WO_3_/NWCNT	Solvothermal process	Precursor: acid-functionalized MWCNTs and Na_2_WO_4_·2H_2_O the structure-directing agent: HNO_3_	Degradation of methylene blue	Degradation percentage of 75% within 150 min	[27]
Graphene/TiO_2_	hydrothermal method	Precursor: GO and TiO_2_ (P25) Time: 24 h Temp:200 °C	Degradation of methylene blue	Degradation percentage of 85% within 100 min	[55]
ZnO-graphene	hydrothermal method	Precursor: GO and the commercial ZnO nanoparticles Time: 12 h Temp:200 °C	Degradation of methylene blue	Degradation percentage of 72.1% within 56 min	[30]
